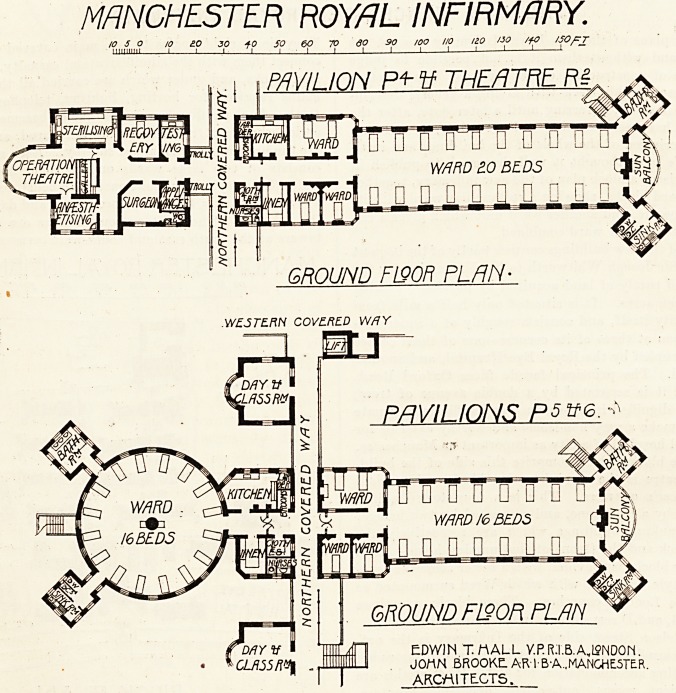# The New Manchester Royal Infirmary

**Published:** 1908-10-24

**Authors:** 


### HOSPITAL ADMINISTRATION.

### CONSTRUCTION AND ECONOMICS.

### THE NEW MANCHESTER ROYAL INFIRMARY.

The full plans of the new Royal Infirmary are not yet available, and without them it is not possible to judge the scheme of construction on its merits. We therefore content ourselves this week with giving a general description of the hospital, deferring until a later date, after the institution has been working a little while, a full and critical description of the whole of the buildings and institution. We have thought it well, however, to publish in the present issue a block plan of the new infirmary, together with two ward blocks, showing a rectangular ward with its operation theatre, and another ward unit which gives a circular and rectangular ward combined.

The site of the new buildings consists partly of the bequest of the late Sir Joseph Whitworth to the University for the purpose, and partly of land acquired by purchase, making in all thirteen acres. It is situated only half a mile from the University itself, and consists roughly of a square encroached upon at three of its corners—one of these corner blocks is occupied by the Royal Eye Hospital, and another by a chapel. The principal façade faces Oxford Road, from which it is separated by a double avenue of trees, and is both dignified in appearance and sufficiently ornate in detail to make a very handsome and worthy exterior for the principal hospital of a city as important as Manchester. Of the three blocks which comprise this side of the buildings, the centre is the administration block and resident medical officer's quarters. This is a four-storied edifice surmounted by a low dome, and connected by two bridges with the flanking buildings which are respectively the teaching block and the (front of the) nursing home. Each of these side blocks is provided with a tower somewhat suggesting in style the types with which Wren surmounted so many of his London churches. These blocks are those marked C, B, and D respectively in the plan reproduced.

On the Nelson Street side of the Infirmary is the outpatient and casualty entrance. Here is a large waiting-room, O, with seating accommodation for 460. West of this are the out-patient rooms, out-patient operating theatres, *x*-ray rooms, and the other usual accessories of a large outpatient department (Block N). Above the casualty department are the gynæcological wards, which, we note, contain but twenty beds, a proportion unduly small considered in relation with the large size of the hospital. The medical and surgical wards are arranged on the pavilion system, and it will be noticed that they occupy the central portion of the site, being thus screened as far as possible from street noises—an advantage not only to the patients themselves, but also to the medical staff, as can be well appreciated by anyone who has tried to do a delicate piece of ausculation amid the rumble of heavy traffic. A special feature of this part of the hospital is the spacious courtyards between and around the pavilions, in which it has been found possible to spare fifty healthy trees from the axe of the builder. In addition to the excellent laboratories and class-rooms in the principal teaching block facing Oxford Road, class-rooms and clinical laboratories are provided in all the units into which the pavilions are subdivided.

Of the ten pavilions, containing in all twenty-five wards, six are allotted to the surgeons and four to the physicians. They lie at right angles to two main covered ways which connect them with the administration, casualty, and admission blocks, and under which are carried all the pipes and mains required for heating, lighting, telephones, and so forth. Each pavilion has an open-paved basement by which aerial separation from the ground is secured, and in which are the branch mains from the subway. Each medical unit consists of two large wards on the same floor, one for males (22 beds) and one for females (18 beds), together with a few small wards, kitchens, store-rooms, and day-rooms for the patients. The sanitary conveniences are in detached towers at the south-east and south-west corners (see plan).

MANCHESTER ROYAL INFIRMARY.

BLOCK PLAN

A denotes west entrance lodge. B, teaching department. C, administration. D K L, female staff home. E, stewards' staff quarters. F, chapel. G, stores. H, servants' dining-room. J, nurses' dining-room. M, north entrance lodge. N, casualty—gynæcological, in-patients, ear, burns, etc. O, out-patients' and pharmacy department. P, surgical pavilions. Q, day rooms. R, operating theatres. S, laundry and workshops. T, medical pavilions. U, physicians' rooms. V, matron's office. W, septic pavilion. X, pathological department. Y, main staircase and lifts. Z, staircases to grounds.

The wards are heated by hot-water radiators arranged in cast-iron casings; these are of a type arranged to fit in with the general scheme of boiler and heating apparatus invented by Mr. E. T. Hall, and the temperature of the circulating water is never more than 160° F., which is said to prevent that "burning" of the air so often detrimentally associated with hot-water heating arrangements. Further devices ensure that in the event of a bursting pipe or similar accident no more than half the radiators in one ward shall be put out of action together.

The surgical wards are individually similar to those of the medical side, but each of the five units contains two male wards of 16 beds each and one female ward of 20 beds.

For each unit there is a separate block (R) to the north of the covered way, containing an operating theatre and its accessories. In these operating theatres are galleries to accommodate students, shut off from the floor of the theatres by glass screens seven feet high.

The hospital, which is now practically complete, contains accommodation for 592 patients and a resident medical, nursing, and administrative staff of about 340. It has been open during the past week for inspection by visitors. Of the 592 beds, 32 are for isolation cases, burns, aural cases, and other special purposes; 20 for gynæcological cases; 300 for surgical and 240 for medical cases. The architects are Mr. E. T. Hall, V.P.R.I.B.A., London, and Mr. John Brooke, A.R.I.B.A., Manchester. The cost of the premises has been about half a million pounds, of which £400,000 was obtained by the sale to the Corporation of the old site in Piccadilly. Towards the remaining £100,000 the sum of £77,000 has already been promised or subscribed, and a great endeavour is being made to secure the remainder before the end of this month; if this is done, Sir W. P. Hartley has offered a subscription of £400 a year for twenty years. The expenses of the Infirmary are estimated as likely to be about £12,000 yearly in excess of those of the old Infirmary, and to the building fund appeal is being added one for additional annual subscriptions. It was hoped that the King would consent to open the new buildings, but his Majesty is unfortunately unable to be present, and it is now not the intention to have any formal opening ceremony.

## Figures and Tables

**Figure f1:**
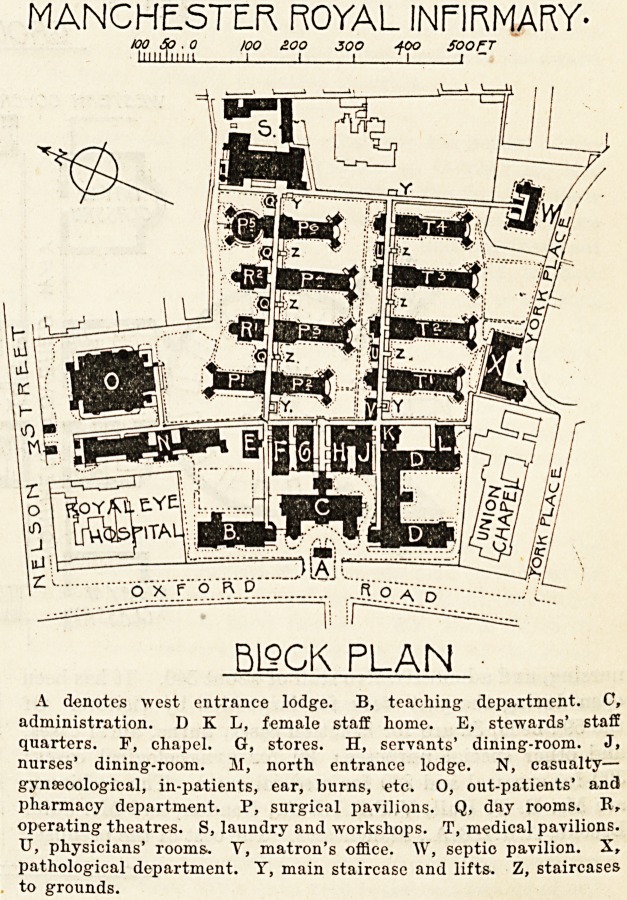


**Figure f2:**